# Exploratory 3D-CT Morphometric Study of the Safe Bony Corridor for Percutaneous Navicular Fixation Across Neutral, Cavus, and Planus Foot Phenotypes

**DOI:** 10.3390/jcm15207796

**Published:** 2026-10-09

**Authors:** Pablo Ruiz-Riquelme, Ricardo Vega-Croker, Daniel Poggio-Cano, Magdalena Castro-Cruz, Mireia Gamundi-López, Ferran Fillat-Gomà, Enrique Adrian Testa

**Affiliations:** 1 Master Fellow Foot and Ankle Surgery, Universitat de Barcelona, 08007 Barcelona, Spain; 2 School of Medicine, Finis Terrae University, Santiago 7501015, Chile; 3 Foot and Ankle Surgery Unit, Hospital Clínico La Florida, Santiago 8242238, Chile; 4 Foot and Ankle Surgery Unit, Clinica Trinitas and Integramedica a part of Bupa Chile, Santiago 7500000, Chile; 5 Foot and Ankle Surgery Unit, Department of Orthopedics and Traumatology, Hospital Clinic de Barcelona, Universitat de Barcelona, 08007 Barcelona, Spain; dpoggio@clinic.cat; 6 Clinical Epidemiology and Research Unit, School of Medicine, Finis Terrae University, Santiago 7501015, Chile; magdalena.castro@uft.cl; 7 3D Surgical Planning Lab, Parc Taulí Hospital Universitari, Institut d’Investigació i Innovació Parc Taulí (I3PT-CERCA), Universitat Autònoma de Barcelona, 08208 Sabadell, Spain; mgamundi@3dptlab.com (M.G.-L.); ffillat@3dptlab.com (F.F.-G.); 8 Orthopedic Department, Parc Taulí Hospital Universitari, Institut d’Investigació i Innovació Parc Taulí (I3PT-CERCA), Universitat Autònoma de Barcelona, 08208 Sabadell, Spain; 9 Service of Orthopaedics and Traumatology, Department of Surgery, EOC, 6500 Bellinzona, Switzerland; 10 Faculty of Biomedical Sciences, Università della Svizzera Italiana (USI), 6900 Lugano, Switzerland; 11 Clinica Ars Medica, Swiss Medical Network, 6929 Gravesano, Switzerland

**Keywords:** navicular bone, foot fractures, bony corridor, osteosynthesis, three-dimensional computed tomography, standardized surgical protocol, foot phenotype, percutaneous fixation

## Abstract

**Background**: The navicular bone is a complex structure with a three-dimensional morphology that varies according to foot phenotype, but has not been completely characterized for safe percutaneous fixation. **Objective**: The objective of this study was to develop and apply a standardized 3D-CT protocol to identify the navicular safe bony corridor and quantify its dimensions according to foot phenotype and sex differences. **Methods**: Using an exploratory cross-sectional observational study, we evaluated 63 navicular bones (21 per phenotype: neutral, cavus, planus), selected by stratified random sampling. A pilot phase was first conducted on nine feet (three per phenotype) to establish and refine the measurement protocol; in this phase, measurements were independently performed by two observers, yielding an excellent inter-observer reliability. The remaining 54 feet were then measured by the biomedical engineer. Comparisons between foot phenotypes and sex were performed using Kruskal–Wallis and Dunn’s post hoc tests with a Bonferroni correction (all two-tailed, α = 0.05). **Results**: The safe bony corridor demonstrated median dimensions of a 38.96 mm length (range: 34.05–47.3 mm), a 10.02 mm diameter (range: 5.84–13.04 mm), and a volume of 3063 mm^3^ (range: 1135–5614 mm^3^). There were no differences in the volume among the phenotypes (*p* = 0.274), although their length differed (*p* = 0.046). The central corridor angle showed significant variation among the phenotypes (*p* = 0.010): cavus, 8.02°; neutral, 7.05°; planus, 6.99°. Corridor occupation varied by phenotype: cavus, 30.9%; neutral, 29.0%; planus, 27.2% (*p* = 0.008). Males demonstrated longer corridors than females (40.14 vs. 37.76 mm, *p* < 0.001), but a similar diameter (*p* = 0.085). The safe diameter (2 mm margin) median was 8.02 mm. **Conclusions**: A standardized and reproducible 3D-CT protocol allows for the precise identification of the safe navicular bony corridor for percutaneous screw fixation with a medial-to-lateral orientation. Although the corridor dimensions remained relatively consistent among phenotypes, the angular and dimensional variability by foot phenotype and sex justifies an individualized preoperative evaluation using 3D-CT to optimize implant selection.

## 1. Introduction

Navicular fractures are uncommon injuries of the midfoot, representing approximately 1–3% of acute midfoot fractures in high-energy trauma and accounting for approximately one-third of foot stress fractures [1,2,3]. Although relatively infrequent, they generate important functional sequelae when managed inadequately [4,5].

The navicular exhibits a complex three-dimensional morphology that varies according to foot phenotype (neutral, cavus, or planus) [6,7]. Recent morphometric studies have confirmed substantial anatomical variation in the navicular shape across individuals, including classification into distinct morphological subtypes [8]. Biomechanically, the navicular transmits a substantial load through the medial longitudinal arch, plays a critical role in the articulation with the talus, and relates to the posterior tibial tendon and medial ligamentous complex [6,9]. The talonavicular articulation permits approximately 80% of hindfoot motion, allowing the hindfoot to flex/lock and the forefoot to supinate/pronate according to the gait phase [9]. Depending on the fracture type and management, patients may develop chronic pain, stiffness, post-traumatic arthritis, or altered foot biomechanics [9,10,11]. Surgical treatment goals include anatomic reduction, the restoration of the medial column length, and early motion enabled by rigid fixation, typically achieved with screws, plates, or both [9,10,12,13].

A critical limitation in navicular osteosynthesis is the lack of a standardized “safe zone” for the percutaneous screw trajectory. Given the navicular’s irregular geometry and proximity to the talonavicular and naviculocuneiform joints, the precise medial-to-lateral screw path remains poorly defined, particularly as it relates to individual variation in foot phenotypes. As weight-bearing CT-based planning tools become increasingly applied in foot and ankle reconstruction [14], the systematic morphometric characterization of safe percutaneous corridors represents a timely methodological framework.

The objective of this study was to develop and apply a standardized three-dimensional computed tomography (3D-CT) protocol to identify the safe bony corridor of the navicular, quantify its dimensions and volume, describe its angular orientation, and secondarily characterize these features according to foot phenotype (neutral, cavus, and planus) and sex.

## 2. Materials and Methods

### 2.1. Study Design and Inclusion Criteria

This was a cross-sectional descriptive exploratory observational study of patients treated for foot and ankle pathology in a public hospital from 2015 to 2019. The study was approved by the institutional research ethics committee (HCB/2022/0078). We included patients aged ≥18 years who has complete high-resolution computed tomography of the foot with a slice thickness ≤ 0.6 mm; a navicular bone without evidence of fracture, arthritis (talonavicular, cuneonavicular, cuboidonavicular), or infection; without implants in adjacent bones generating artifacts; and with a documented clinical and/or radiographic foot phenotype (cavus, neutral, or planus). We excluded cases with a history of tarsal surgery, the presence of metallic implants generating artifacts, or navicular bones with destructive osseous lesions. From the eligible cohort, the patients were stratified by foot phenotype, and 21 feet per phenotype stratum were selected by simple random sampling using a random-number generator (www.random.org), yielding *n* = 63 specimens for the analysis.

### 2.2. Demographic Data and CT Protocol

Age, sex, foot laterality, and foot phenotype (neutral, cavus, or planus) were recorded from clinical records. Phenotype classification was determined by a fellowship-trained foot and ankle surgeon with more than 20 years of clinical and surgical experience, based on a combined clinical examination (arch assessment, hindfoot alignment, forefoot position) and weight-bearing radiographic assessment. The following radiographic indices were used: Meary’s angle, the calcaneal pitch, the anteroposterior and lateral talocalcaneal angles, the talar declination, the navicular coverage percentage, and the Saltzman view. Classification was performed for the purpose of the original clinical care episode, prior to study inclusion.

A 128-detector CT scanner (SOMATOM Definition Edge, Siemens AG, Munich, Germany) was used with the following parameters: a 0.6 mm slice thickness, a bone reconstruction algorithm, a standard bone kernel, an FOV = 200 mm, and a matrix of 512 × 512. All acquisitions were performed with the patient in supine position with a neutral foot alignment (non-weight-bearing).

### 2.3. Measurement Protocol and Variables Evaluated

Eleven morphometric variables were measured using 3D reconstruction software (Slicer-3D v.4.11) and processing software (3-Matic v.18.0, Materialize NV, Leuven, Belgium) from anonymized DICOM files. A pilot phase was first conducted on 9 feet (3 per phenotype: neutral, cavus, and planus) to develop and refine the measurement protocol by consensus between a biomedical engineer and an orthopedic traumatologist with specialist expertise in 3D modeling. In this pilot phase, both observers independently performed all measurements, blinded to each other’s results, to evaluate the inter-observer reproducibility (Appendix A). Following a satisfactory reliability assessment, the standardized protocol was applied to all 63 specimens; the remaining 54 feet were measured exclusively by the biomedical engineer.

**Figure 1:**
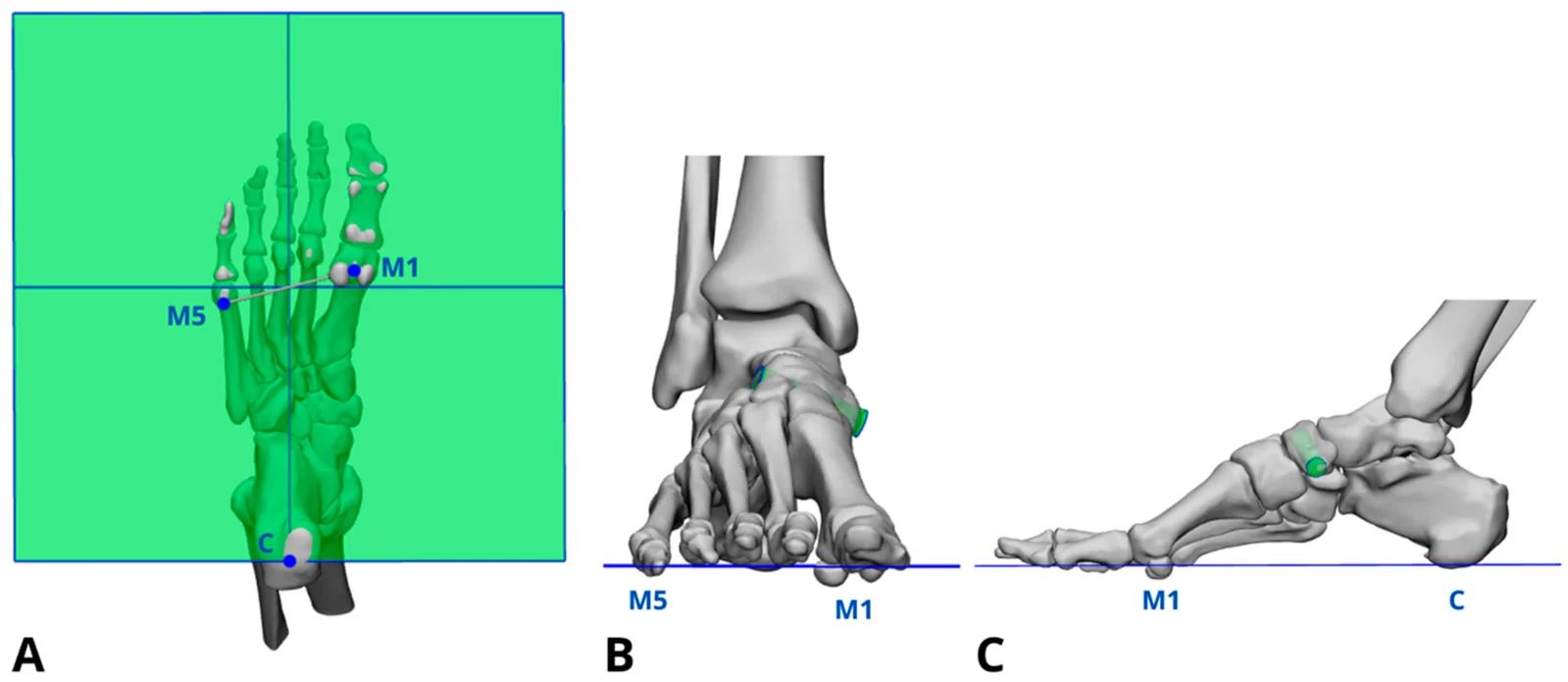
Triangular support plane of 3D reconstruction of a right foot. Triangle based on three points, one on the first metatarsal head (M1), one on the fifth metatarsal head (M5) and one on the lowest point of the calcaneus (C). 3D reconstruction foot views: (**A**) plantar, (**B**) anteroposterior and (**C**) medial.

Each three-dimensional foot was first positioned on a standardized triangular support plane. Three landmark points were programmed: at the heads of the first and fifth metatarsals and at the lowest point of the calcaneus [15,16] (Figure 1). The support plane of the navicular bone was determined in a coronal plane by a line joining the two most prominent plantar points of the bone. The navicular was then isolated for analysis.

**Figure 2:**
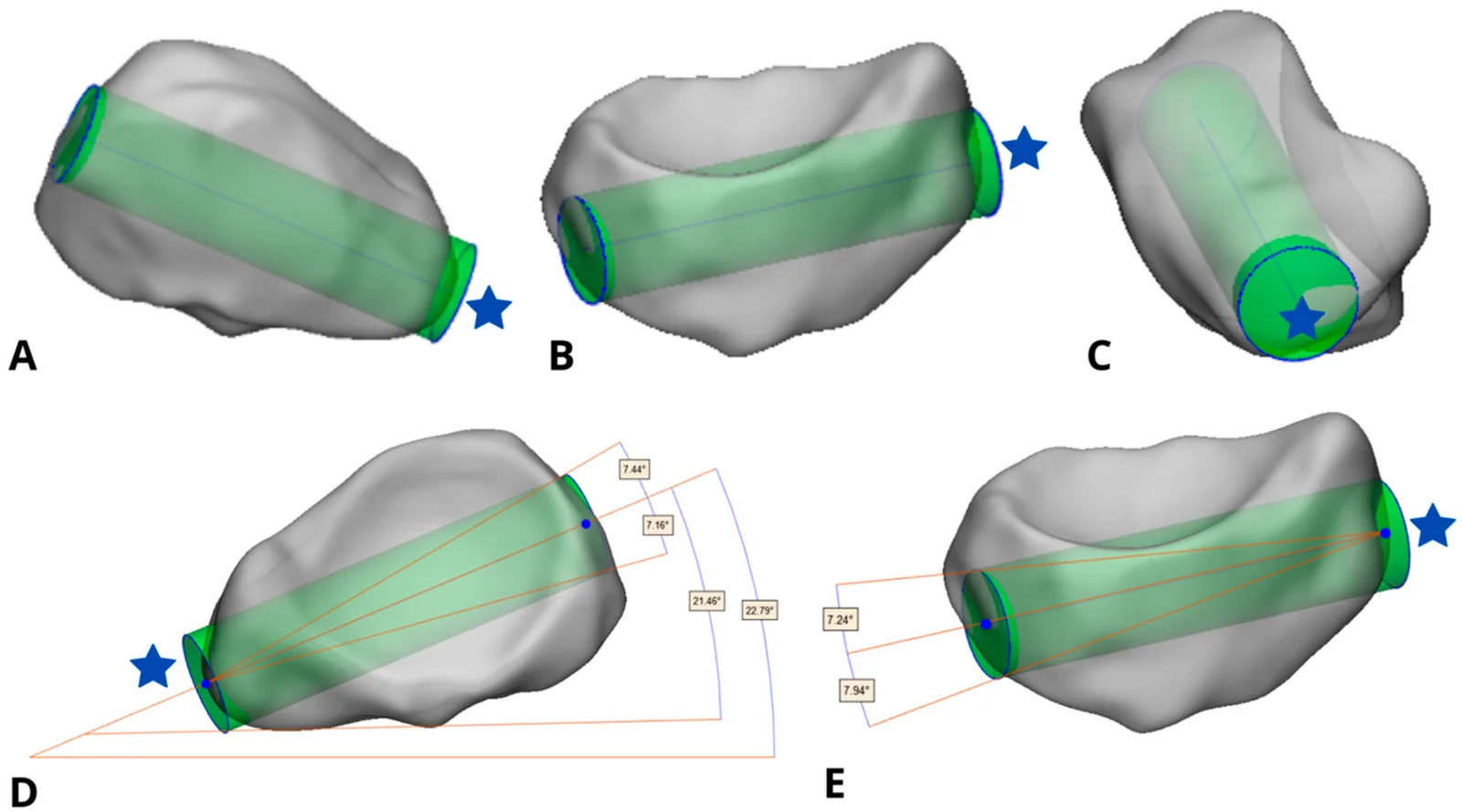
Maximum cylinder inside the navicular (navicular bone corridor run), and coronal and axial angulations of the central axis of the corridor. Views anterior to the cuneiform facet (**A**), superior oblique (**B**) and medial oblique (**C**) of the bony corridor. Maximum angulation measurements in the coronal (**D**) and axial (**E**) planes of the central axis of the bony corridor. The blue star indicates the medial aspect of the navicular.

To determine the safe bony corridor, the largest intraosseous cylinder was identified. The main axis was defined as the line of greatest distance in the coronal plane, extending from the most medial and prominent aspect to the most lateral aspect of the navicular. A cylinder was then programmed along this axis and expanded to define the maximum corridor size before exiting the bone or entering the articular zone (Figure 2). The cylinder had a circular cross-section; consequently, the height and width of the corridor were geometrically equivalent and were reported as a single diameter value. The angulation of the bony corridor in the coronal and axial plane was evaluated in relation to the plane of support of the foot and the plane of the navicular. This represented the maximum angulation of the screw at its medial-to-lateral entry point (Figure 2). We evaluated the total navicular volume, safe bony corridor volume, corridor length (sagittal axis), corridor diameter (circular cross-section, single value), maximum axial angles (anterior and posterior), coronal angles relative to the navicular base, coronal angles relative to the foot base, and maximum coronal angles (dorsal and plantar).

### 2.4. Definition of Safe Bony Corridor

We defined the safe bony corridor as the bony space available with a 1 mm reduction in the cylinder radius (equivalent to a 2 mm reduction in diameter) from the maximum inscribed cylinder, applied to maintain a minimum distance from the cortical edges and articular cartilage. This margin is consistent with the safety distances applied in analogous percutaneous fixation corridors in anatomically constrained bones such as the acetabulum [17] and the carpal scaphoid [18], where 1–2 mm from the cortical edges is considered sufficient to accommodate minor intraoperative trajectory deviations during cannulated screw insertion. The screws used in navicular fracture fixation range from a 2.0 mm diameter for small fragment fixation to a 3.5–4.5 mm diameter for navicular body fractures, with 2.7 mm cortical screws and 3.5–4.0 mm cannulated screws most commonly used according to the fracture pattern and fragment size [9,10,12,13].

### 2.5. Statistical Analysis

This exploratory study evaluated a stratified random sample of 63 feet, 21 per phenotype, selected as described in the previous section, consistent with the sample sizes reported in the morphologic literature for navicular bone studies [7,19,20,21].

The inter-observer reliability was evaluated in the pilot phase only (9 feet; 3 per phenotype). Both observers (a biomedical engineer and an orthopedic surgeon) independently measured all 10 variables in this subset, blinded to each other’s results. Intraclass correlation coefficients (ICC [1,2], two-way mixed-effects model, absolute agreement, single measures) were calculated. The ICC values ranged from 0.933 to 0.940 across all variables, indicating excellent inter-observer reliability [22] (Appendix A Table A1). The remaining 54 feet were measured by the biomedical engineer only, who was a specialist in 3D modeling; the intra-observer reliability for the full sample was not formally assessed and is acknowledged as a study limitation.

The analysis was conducted using Stata v.14. Comparisons between foot phenotypes and sex were performed using the Kruskal–Wallis test (nonparametric for three groups), followed by pairwise Dunn comparisons with a Bonferroni correction when appropriate (all two-tailed, α = 0.05). A post hoc power analysis using the observed effect sizes yielded: Cohen’s d = 1.32 for navicular volume by sex (power ≥ 95%); Cohen’s f = 0.74 for plantar angular excursion by phenotype (power ≥ 95%); and Cohen’s f = 0.33 for corridor length by phenotype (power ≈ 50%; underpowered—reported as exploratory finding). The data are expressed as the median (range: minimum–maximum) throughout.

## 3. Results

Sixty-three navicular bones were included: 62% (39/63) male and 38% (24/63) female, with a median age of 51 years (range: 19–75) and 55.6% (35/63) right-sided. There were 21 feet for each phenotype (neutral, cavus, planus). No significant age differences were found by phenotype (*p* = 0.187) or sex (*p* = 0.156). The age and laterality were not significantly associated with the corridor orientation or dimensions (*p* > 0.20), and the corridor volume and dimensions were equivalent between lateralities.

### 3.1. Navicular Bone Volume

The median navicular bone volume was 10,942.13 mm^3^ (range: 5504.87–15,944.50) or 10.94 cm^3^.

### 3.2. Navicular Bony Corridor—Dimensions and Occupation Ratio

The median bony corridor volume was 3063.28 mm^3^ (range: 1135.11–5613.98) or 3.06 cm^3^, with a median length of 38.96 mm (range: 34.05–47.3 mm) and a median width/height of 10.02 mm (range; 5.84–13.04 mm). The corridor occupied a median of 29.4% (range: 8.6–52.4%) of the total navicular bone volume. This ratio did not differ by sex, but differed significantly by phenotype (Table 1), with cavus feet demonstrating the largest corridor occupancy at 30.9% (range: 24.1–52.4%), neutral feet at 29.0% (range: 9.1–35.2%), and planus feet at 27.2% (range: 8.6–36.7%) (*p* = 0.008).

Males had a significantly greater navicular volume (*p* < 0.001), corridor volume (*p* = 0.014), and corridor length (*p* < 0.001) than females, with no significant differences in the corridor diameter or angular orientation (Table 2. A shorter corridor length was found overall in cavus versus other phenotypes (*p* = 0.046); however, no individual pairwise comparison survived the Bonferroni correction (cavus versus neutral *p*_adj = 0.091; cavus versus planus *p*_adj = 0.100; neutral versus planus *p*_adj = 1.000). This corridor-length difference should be interpreted as an exploratory finding, given insufficient statistical power for this comparison. A smaller total angular range in the coronal plane was found in cavus feet (dorsal *p* = 0.004; plantar *p* < 0.001) versus other phenotypes.

Males had a significantly greater navicular volume and bony corridor volume (*p* < 0.001 and *p* = 0.014, respectively) and a longer bony corridor (*p* < 0.001) than females, with no significant differences in the corridor diameter or angular orientation (Table 2). A shorter bony corridor length was found in cavus feet versus other phenotypes (*p* = 0.046) (Table 2). A smaller total range of angulation in the coronal plane (dorsal and plantar) for the corridor bone axis was found in cavus feet versus other phenotypes (*p* = 0.030 and *p* = 0.001).

The slenderness index (length-to-width ratio) had a median value of 3.94 (range: 2.88–7.66), indicating that the corridor is approximately 3.9 times longer than its diameter. This index varied by phenotype: neutral feet, 3.95; cavus feet, 3.57 (more compact); and planus feet, 4.16 (more slender). The safe diameter of the corridor (height/width minus the 2 mm safety margin) had a median value of 8.02 mm (range: 6.2–10.8 mm), confirming compatibility with standard percutaneous implants of a 2.0–4.0 mm diameter with an adequate cortical margin.

### 3.3. Navicular Bony Corridor—Angular Orientation

The median coronal inclination of the bony corridor axis in relation to the reference surface was 7.35° and 7.51° (Table 3). The total range of motion of the bony corridor axis (screw medial position) was 34° and 16° in the coronal and axial planes, respectively (Table 4). Significant variation in coronal angulation was found among foot phenotypes. The maximum plantar inclination angle differed between neutral feet at 22.06°, cavus feet at 9.46°, and planus feet at 27.34° (*p* < 0.001) (Table 5), representing an 18° difference between cavus and planus feet. The dorsal inclination angle also varied significantly (*p* = 0.004) (Table 6): neutral, 17.00°; cavus, 11.45°; and planus, 8.64°. These phenotype-dependent differences in corridor orientation necessitate individualized surgical planning based on a preoperative 3D-CT evaluation.

### 3.4. Coefficient of Variation and Variability Analysis

The calculation of the coefficient of variation (CV) for key parameters revealed low variability in the dimensional measurements (corridor length CV = 7.9%, corridor diameter CV = 14.9%), indicating highly predictable linear dimensions across the cohort. The coronal central axis angle of the navicular bony corridor, measured relative to the foot’s triangle of support, demonstrated moderate variability (CV = 16.33%), with significant differences by phenotype: cavus feet showed more vertical orientation (8.02°, CV = 12.76%), while planus (*p* = 0.020) and neutral (*p* = 0.004) feet showed more horizontal orientation (6.99°, CV = 18.92% and 7.05°, CV = 13.95%, respectively).

## 4. Discussion

The navicular bone plays a critical role in the medial arch of the foot, as its articulation with the talus permits approximately 80% of the hindfoot [9]. Although fractures of this bone are uncommon, they generate important functional sequelae when managed inadequately. One of the least standardized technical aspects is the safe trajectory of the percutaneous screw through the navicular body, given its irregular geometry [7,19,23,24] and proximity to the talonavicular and naviculocuneiform joints [6].

**Figure 3:**
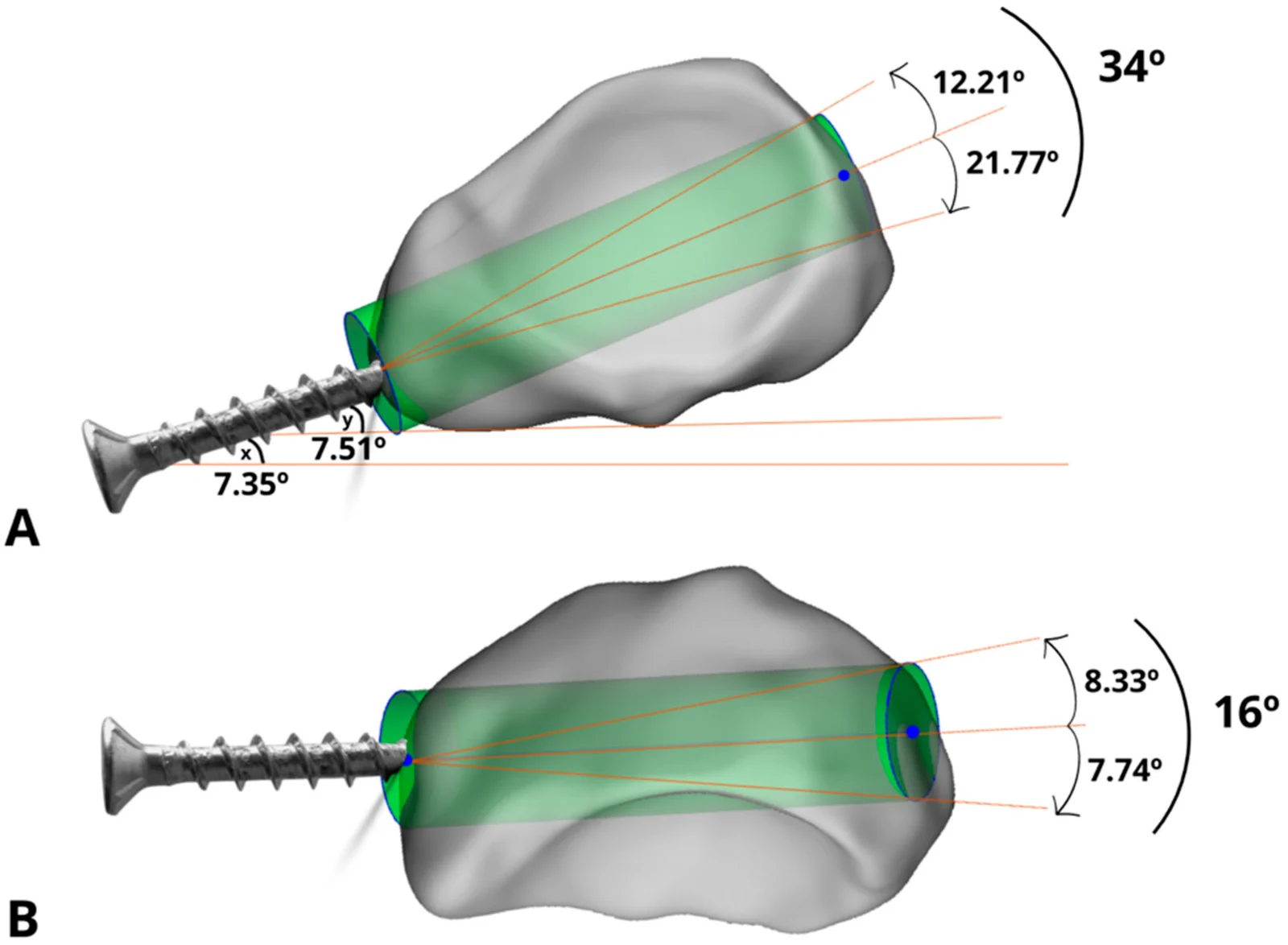
Clinical application of the navicular’s bone corridor run. Screw with entry in medial tuberosity of the navicular with medial–lateral direction in coronal plane, with an angulation of 7.35° with respect to the support plane of the foot triangle or 7.51° with respect to the plantar plane of the navicular (**A**). Median range of motion in coronal plane of 12.21° dorsal and 21.77° plantar with 34° total range of motion (**A**). Median range of motion in axial plane of 8.33° anterior and 7.74° posterior with 16° total range of motion (**B**).

Our study characterized the navicular bony corridor using quantitative three-dimensional measurements, establishing its dimensions and angular variability according to foot phenotype, thereby facilitating the planning of osteosynthesis with percutaneous screw fixation in the medial-to-lateral coronal direction (Figure 3). The navicular bony corridor demonstrated median dimensions of a 38.96 mm length, a 10.02 mm diameter, and a volume of 3063 mm^3^, indicating a relatively narrow surgical space requiring precise planning. We found that the central corridor angle varied significantly by phenotype (*p* = 0.010), ranging generally between 7° and 8° relative to the foot’s weight-bearing triangle. To our knowledge, no previous study has specifically quantified a standardized intraosseous corridor for percutaneous navicular fixation. Several morphometric studies have described relevant aspects of navicular anatomy. Ao et al. [8] analyzed 351 CT-based navicular reconstructions and classified the navicular into three morphological shapes, finding significant sex-based differences in all measurements except one angular parameter, consistent with our finding of a greater navicular and corridor volume in males. Moore et al. [7] reported that cavus feet display a shorter, more compact navicular with a greater lateral volume, consistent with the shorter corridor we observed in cavus phenotypes (median: 37.2 mm versus 40.3 mm in neutral, *p* = 0.046). Peeters et al. [19] described a more proximally oriented talonavicular facet in planus feet, and Louie et al. [20] found no differences in the navicular height-to-width ratio between phenotypes, consistent with our finding of no significant phenotypic difference in the corridor diameter. Wahlberg et al. [24] reported a more inverted talonavicular joint in cavus feet, which may explain the reduced plantar angular excursion we observed in this phenotype (median: 9.5° versus 22.1° in neutral and 27.3° in planus, *p* < 0.001). Our data extended this literature by providing the first quantitative characterization of the safe intraosseous corridor across phenotypes. Emerging robot-assisted systems for percutaneous navicular fixation [25] further underscore the clinical need for precise preoperative corridor data. Based on these preliminary anatomical findings, cavus feet may benefit from planning shorter implants and careful trajectories; planus feet should always be evaluated preoperatively with 3D-CT and reconstructed images before fixation, while neutral feet allow more flexibility, though the verification of individual variability remains prudent.

Sex-related differences in the navicular morphology have been previously described. Saldias et al. [23] found differences in the maximum height of its size and in the length of the talar and cuneiform facets. Moore et al. [7] found that males have a lower and more recessed prominence, while females have a smoother lower prominence. In our sample, the bony corridor was significantly longer in males (40.14 mm) than females (37.76 mm), *p* < 0.001, with a greater available volume (*p* = 0.014), but without differences in the corridor diameter (males: 10.11 vs. females: 9.67 mm; *p* = 0.085) or central corridor angulation (males: 7.25° vs. females: 7.52°; *p* = 0.203). These findings indicate that, while the navicular and corridor size vary by sex, the angular properties of the corridor remain consistent between males and females, allowing for the generalization of angle-based surgical techniques across sexes.

The safety of percutaneous fixation depends on the fracture type and the availability of a bony corridor in that specific fragment. Sangeorzan [26] classifies navicular fractures into three types: type I (sagittal), type II (central with comminution), and type III (complete comminution). In type I and II fractures, where an adequate central fragment persists, our measurements indicated that a 3.5–4.5 mm diameter screw can be safely positioned within the defined margin. In type III fractures with substantial comminution, the bony corridor is significantly reduced and smaller screws (2.7–3.5 mm) may be necessary in association with plate fixation [27,28]. Regarding the screw diameter, we propose a practical analysis that takes into account a safety margin between the canal and the screw, arbitrarily defining as a safe zone those screws that maintain a total distance of at least 2 mm between the diameter of the bony corridor (generally in the coronal plane) and the margins of the navicular bone (Table 7 and Table 8).

This study had limitations. It was a descriptive observational design that characterized the anatomy, volume, and bony corridor of the navicular; it did not validate the clinical outcomes of actual fixation. Although we demonstrated statistically significant phenotypic differences in the angular corridor orientation (*p* ≤ 0.004), the corridor-length difference by phenotype (*p* = 0.046) should be interpreted as an exploratory finding, given the insufficient statistical power for that comparison. The population was of Mediterranean origin with a modest sample size (21 per phenotype). Importantly, the inter-observer reliability was evaluated in the pilot phase only (nine feet; three per phenotype); the intra-observer reliability for the full sample of 54 feet measured by a single observer was not formally assessed. A formal ICC assessment across the entire 63-foot sample was not performed, and this constitutes a limitation of the present study. Future studies should evaluate both the inter- and intra-observer reliability across the full sample. This study was conducted in unfractured navicular bones and did not evaluate the actual screw placement accuracy, cortical breach rates, construct stability, or clinical outcomes. The safety corridor described here reflects anatomical feasibility, not demonstrated surgical safety. Future biomechanical validation in cadaveric models and prospective clinical series are required before these morphometric data can be directly translated into surgical protocols. Additionally, phenotype classification was performed by a single experienced surgeon; future studies should evaluate inter-observer agreement for phenotype assignment using quantitative radiographic indices. Despite these limitations, these findings establish the foundation for specific surgical protocols, reducing the risk of inadvertent articular compromise.

The clinical implication of this study is the definition of a bony corridor for the navicular bone, which facilitates the orientation and sizing of the implant, considering the patient’s sex and phenotype. These morphometric data may inform preoperative planning. According to the Sangeorzan fracture classification, type I fractures have an intact corridor permitting standard 3.5–4.5 mm screw fixation; however, the screw diameter should be considered based on the fracture size and location, for example, 2.0 to 2.7 for small fragments. For type II fractures, the ideal corridor scenario, a preoperative evaluation via 3D-CT is warranted to confirm corridor availability and select the implant size (Table 8). In type III fractures, the corridor is severely compromised; bridge plate fixation alternatives should be considered if the congruence around percutaneous screw fixation is not achievable. According to foot phenotype variability, planus feet are the most variable and demand preoperative imaging certainty if associated with type II–III fractures.

## 5. Conclusions

This exploratory observational study defined and morphometrically characterized a navicular bony corridor permitting its percutaneous fixation with a medial-to-lateral screw orientation. The central corridor axis was oriented 7–8° relative to the foot’s plantigrade support; however, its angular range was variable according to the foot phenotype, and an individualized preoperative assessment is warranted. The corridor volume was relatively consistent among the phenotypes and greater in males than females. Cavus feet were more predictable, but more constrained; planus feet require individualization. Quantification via preoperative 3D-CT may support the optimization of implant selection and the trajectory according to the phenotype and fracture type. These findings were derived from the morphometry of unfractured bones and constitute preliminary anatomical proposals. Clinical studies validating screw-positioning accuracy, cortical breach rates, and complication rates are required before these findings can be recommended as surgical standards.

## Figures and Tables

**Table 1:** Bony corridor occupation ratio and geometric indices by foot phenotype.

Geometric Measure	Neutral (*n* = 21) Median (Range)	Cavus (*n* = 21) Median (Range)	Planus (*n* = 21) Median (Range)	*p*-Value ^a^
Corridor Occupation Ratio (%) ^b^	29.0 (9.1–35.2)	30.9 (24.1–52.4)	27.2 (8.6–36.7)	0.008 *
Slenderness Index (L/W) ^c^	3.95 (3.46–7.66)	3.57 (2.88–4.65)	4.16 (3.34–7.00)	0.006 *
Clinical Safe Diameter (1 mm radius margin) (mm)	8.02 (6.2–10.8)	8.15 (6.5–10.3)	7.85 (6.2–10.8)	—

L/W, length-to-width ratio. ^a^ Kruskal–Wallis test (overall group comparison). ^b^ Corridor occupation ratio—post hoc Dunn (Bonferroni): cavus > planus *p*_adj = 0.008; other pairs not significant. ^c^ Slenderness index—post hoc Dunn (Bonferroni): neutral > cavus *p*_adj = 0.023; cavus < planus *p*_adj = 0.014; neutral vs. planus *p*_adj = 1.000. * *p* < 0.05. Descriptive measure only; not statistically compared.

**Table 2:** Evaluation of navicular volume and navicular’s bony corridor run (volume; dimensions; angulations) according to sex, age and foot phenotype.

**A.** Navicular’s bony corridor by sex.					
**Variable**	**Female (*n* = 24) Median (Range)**		**Male (*n* = 39) Median (Range)**		***p*-Value ^a^**
**Navicular**					
Volume (mm^3^)	9063 (5505–12,660)		11,704 (7592–15,944)		<0.001 *
**Navicular’s bony corridor**					
Volume (mm^3^)	2598 (1249–5048)		3278 (1135–5614)		0.014 *
*Dimensions (mm)*					
Length	37.1 (34.1–47.3)		40.4 (35.6–44.7)		<0.001 *
Height or width	9.7 (6.3–13.0)		10.3 (5.8–13.0)		0.084
*Angulation—median (°)*					
Coronal plane, dorsal	13.5 (0.25–30.8)		11.9 (2.38–38.0)		1.000
Coronal plane, plantar	23.1 (4.2–45.4)		21.0 (1.5–50.6)		0.488
Axial plane, anterior	8.6 (5.3–11.2)		8.2 (4.4–11.6)		0.357
Axial plane, posterior	7.8 (5.1–9.5)		7.5 (4.3–10.5)		0.453
Values are expressed as median (range: minimum–maximum). ^a^ Mann–Whitney U test. * *p* < 0.05.					
**B.** Navicular’s bony corridor by age.					
**Variable**	**<50 Years (*n* = 30) Median (Range)**		**≥50 Years (*n* = 33) Median (Range)**		***p*-Value ^a^**
**Navicular**					
Volume (mm^3^)	11,353 (5504–15,944)		10,260 (6984–15,318)		0.123
**Navicular’s bony corridor**					
Volume (mm^3^)	3196 (1622–5614)		2812 (1135–4886)		0.321
*Dimensions (mm)*					
Length	37.7 (35.1–47.3)		38.1 (34.1–44.7)		0.482
Height or width	10.3 (7.6–13.0)		9.9 (5.8–13.0)		0.360
*Angulation—median (°)*					
Coronal plane, dorsal	13.7 (0.25–27.7)		11.4 (5.4–38.0)		0.639
Coronal plane, plantar	17.3 (1.5–50.6)		25.3 (1.6–54.4)		0.013 *
Axial plane, anterior	8.4 (5.3–11.2)		8.3 (4.4–11.6)		0.572
Axial plane, posterior	7.7 (5.1–9.4)		7.6 (4.3–10.5)		0.526
Values are expressed as median (range: minimum–maximum). ^a^ Mann–Whitney U test. * *p* < 0.05.					
**C.** Navicular’s bony corridor by phenotype.					
**Variable**	**Neutral (*n* = 21) Median (Range)**	**Cavus (*n* = 21) Median (Range)**		**Planus (*n* = 21) Median (Range)**	***p*-Value ^a^**
**Navicular**					
Volume (mm^3^)	11,062 (7592–15,944)	10,601 (5504–12,781)		10,860 (7568–15,416)	0.451
**Navicular’s bony corridor**					
Volume (mm^3^)	2868 (1191–5614)	3129 (1622–5048)		2919 (1135–4668)	0.274
*Dimensions (mm)*					
Length ^b^	40.3 (35.0–44.7)	37.2 (34.1–43.0)		39.5 (34.5–47.3)	0.046 *
Height or width	9.9 (5.8–12.7)	10.3 (7.6–13.0)		9.8 (5.9–12.0)	0.105
*Angulation—median (°)*					
Coronal plane, dorsal ^c^	17.00 (8.6–38.0)	11.45 (0.25–30.8)		8.64 (5.4–24.4)	0.004 **
Coronal plane, plantar ^d^	22.06 (13.4–46.1)	9.46 (1.6–29.2)		27.34 (1.5–54.4)	<0.001 ***
Axial plane, anterior	8.1 (4.4–9.8)	8.5 (6.5–11.6)		8.4 (4.7–10.4)	0.394
Axial plane, posterior	7.4 (4.6–9.1)	7.9 (6.2–10.5)		7.8 (4.3–9.5)	0.251
Values are expressed as median (range: minimum–maximum). ^a^ Kruskal–Wallis test. ^b^ Overall *p* = 0.046; no pairwise comparison survived Bonferroni correction (Dunn’s test): Neutral vs. cavus *p*_adj = 0.091; cavus vs. planus *p*_adj = 0.100; neutral vs. planus *p*_adj = 1.000. Exploratory finding. ^c^ Post hoc Dunn (Bonferroni): neutral > planus *p*_adj = 0.004; other pairs not significant. ^d^ Post hoc Dunn (Bonferroni): cavus < neutral *p*_adj = 0.001; cavus < planus *p*_adj < 0.001; neutral vs. planus *p*_adj = 0.855. * *p* < 0.05; ** *p* < 0.01; *** *p* < 0.001.					

**Table 3:** Maximum angulation of the bony corridor’s central axis in the coronal and axial planes.

Plane/Direction	Median (Range) (°)	Total ROM (°)
Coronal plane, dorsal	12.21 (0.25–37.91)	34
Coronal plane, plantar	21.77 (1.48–54.41)	
Axial plane, anterior	8.33 (4.40–11.66)	16
Axial plane, posterior	7.74 (4.27–10.48)	

ROM, range of motion.

**Table 4:** Coronal axis inclination of the bony corridor relative to the foot support triangle and navicular base by foot phenotype.

Variable	Neutral (*n* = 21) Median (Range) (°)	Cavus (*n* = 21) Median (Range) (°)	Planus (*n* = 21) Median (Range) (°)	*p*-Value ^a^
Coronal axis vs. foot support triangle ^b^	7.05 (3.66–9.35)	8.02 (5.01–10.16)	6.99 (3.94–10.28)	0.010 *
Coronal axis vs. navicular base	7.35 (3.90–9.66)	8.15 (5.10–10.50)	7.15 (4.12–10.28)	0.087

Values are expressed as median (range: minimum–maximum). ^a^ Kruskal–Wallis test. ^b^ Post hoc Dunn (Bonferroni): cavus > neutral *p*_adj = 0.021; cavus > planus *p*_adj = 0.033; neutral vs. planus *p*_adj = 1.000. * *p* < 0.05.

**Table 5:** Maximum coronal plantar angulation of the bony corridor by foot phenotype.

Foot Phenotype	Median (°)	Minimum (°)	Maximum (°)	*n*
Neutral	22.06	13.38	46.10	21
Cavus	9.46	1.59	29.16	21
Planus	27.34	1.48	54.41	21

Kruskal–Wallis *p* < 0.001. Post hoc Dunn (Bonferroni): cavus < neutral *p*_adj = 0.001; cavus < planus *p*_adj < 0.001; neutral vs. planus *p*_adj = 0.855.

**Table 6:** Maximum coronal dorsal angulation of the bony corridor by foot phenotype.

Foot Phenotype	Median (°)	Minimum (°)	Maximum (°)	*n*
Neutral	17.00	8.59	37.91	21
Cavus	11.45	0.25	30.81	21
Planus	8.64	5.44	24.35	21

Kruskal–Wallis *p* = 0.004. Post hoc Dunn (Bonferroni): neutral > planus *p*_adj = 0.004; other pairs not significant.

**Table 7:** Navicular volume and bony corridor dimensions by sex.

Variable	Female (*n* = 24) Median (Range)	Male (*n* = 39) Median (Range)	*p*-Value ^a^
Navicular volume (mm^3^)	9063 (5505–12,660)	11,704 (7592–15,944)	<0.001 ***
Corridor volume (mm^3^)	2598 (1249–5048)	3278 (1135–5614)	0.014 *
Corridor length (mm)	37.1 (34.1–47.3)	40.4 (35.6–44.7)	<0.001 ***
Corridor diameter (mm)	9.7 (6.3–13.0)	10.3 (5.8–13.0)	0.084

Values are expressed as median (range: minimum–maximum). ^a^ Mann-Whitney U test. * *p* < 0.05; *** *p* < 0.001.

**Table 8:** Screw options based on the safety margin (safety margin: canal diameter − screw diameter) according to the navicular bone corridor diameter for foot phenotype, sex, and both.

**A.** Based on phenotype (median).							
**Phenotype**	**Navicular Corridor Diameter (mm)**	**Safety Margin (mm) = Navicular Corridor Diameter − Screw Diameter**					
		**1 × 3.5 mm**	**1 × 4.5 mm**	**2 × 2.7 mm ^a^ (sep. 2 mm)**	**2 × 3.0 mm ^a^ (sep. 2 mm)**	**2 × 2.7 mm ^a^ (sep. 3 mm)**	**2 × 3.0 mm ^a^ (sep. 3 mm)**
**Cavus**	10.32	6.82	5.82	2.92	2.32	1.22 *	1.32 *
**Neutral**	9.90	6.40	5.40	2.50	1.90 *	0.80 *	0.90 *
**Planus**	9.82	6.32	5.32	2.42	1.82 *	0.72 *	0.82 *
^a^ Two screws placed with the stated separation. * Safety margin < 2 mm—risk of the screw exiting the bone (defined as a margin of less than 2 mm between the screw diameter, in the coronal plane, and the margins of the navicular bone corridor).							
**B.** Based on sex (median).							
**Sex**	**Navicular Corridor Diameter (mm)**	**Safety Margin (mm) = Navicular Corridor Diameter − Screw Diameter**					
		**1 × 3.5 mm**	**1 × 4.5 mm**	**2 × 2.7 mm ^a^ (sep. 2 mm)**	**2 × 3.0 mm ^a^ (sep. 2 mm)**	**2 × 2.7 mm ^a^ (sep. 3 mm)**	**2 × 3.0 mm ^a^ (sep. 3 mm)**
**Male**	10.30	6.80	5.80	2.90	2.30	1.20 *	1.30 *
**Female**	9.69	6.19	5.19	2.29	1.69 *	0.59 *	0.69 *
^a^ Two screws placed with the stated separation. * Safety margin < 2 mm—risk of the screw exiting the bone.							
**C.** Based on phenotype and sex (median).							
**Phenotype + Sex**	**Navicular Corridor Diameter (mm)**	**Safety Margin (mm) = Navicular Corridor Diameter − Screw Diameter**					
		**1 × 3.5 mm**	**1 × 4.5 mm**	**2 × 2.7 mm ^a^ (sep. 2 mm)**	**2 × 3.0 mm ^a^ (sep. 2 mm)**	**2 × 2.7 mm ^a^ (sep. 3 mm)**	**2 × 3.0 mm ^a^ (sep. 3 mm)**
**Cavus + Male**	10.43	6.93	5.93	3.03	2.43	1.33 *	1.43 *
**Cavus + Female**	10.21	6.71	5.71	2.81	2.21	1.11 *	1.21 *
**Neutral + Male**	10.04	6.54	5.54	2.64	2.04	0.94 *	1.04 *
**Neutral + Female**	9.56	6.06	5.06	2.16	1.56 *	0.46 *	0.56 *
**Planus + Male**	9.82	6.32	5.32	2.42	1.82 *	0.72 *	0.82 *
**Planus + Female**	9.25	5.75	4.75	1.85 *	1.25 *	0.15 *	0.25 *
^a^ Two screws placed with the stated separation. * Safety margin < 2 mm—risk of the screw exiting the bone.							

## Data Availability

There is no additional information to provide beyond what is published in this article. For data-protection reasons, only the researchers involved in this study may access it.

## Abbreviations

The following abbreviations are used in this manuscript:

3D-CT	Three-dimensional computed tomography
3D	Three-dimensional
CT	Computed tomography

## Appendix A

**Table A1:** Inter-observer reliability—pilot phase (*n* = 9 feet; 3 per phenotype). Both observers independently measured all 10 morphometric variables in 9 specimens, blinded to each other’s results. ICC [1,2] (two-way mixed-effects model, absolute agreement) was calculated following the guidelines of Koo and Li [22].

Variable	Observer 1 Median [IQR]	Observer 2 Median [IQR]	ICC (2,1) (95% CI)	Interpretation ^a^
Navicular volume (mm^3^)	10,601.1 [9797.7–12,683.5]	10,633.0 [8945.1–11,724.1]	0.937 [0.778–0.987]	Excellent
Bony corridor volume (mm^3^)	3083.4 [2607.0–3811.5]	3110.5 [2778.1–3779.5]	0.940 [0.734–0.985]	Excellent
Corridor length (mm)	39.27 [37.14–40.38]	39.31 [38.01–39.79]	0.936 [0.774–0.987]	Excellent
Corridor diameter (mm)	10.24 [9.44–10.52]	10.36 [9.57–10.50]	0.940 [0.733–0.984]	Excellent
Max axial anterior angulation (°)	8.30 [7.16–8.83]	8.42 [7.61–9.14]	0.934 [0.804–0.989]	Excellent
Max axial posterior angulation (°)	7.75 [6.79–8.09]	7.87 [7.17–8.31]	0.935 [0.808–0.989]	Excellent
Coronal axis vs. navicular base (°)	7.79 [7.13–7.93]	7.86 [7.20–7.97]	0.939 [0.818–0.990]	Excellent
Coronal axis vs. foot base (°)	7.54 [6.86–7.74]	7.66 [7.05–8.19]	0.936 [0.809–0.989]	Excellent
Max coronal dorsal angulation (°)	13.16 [7.53–14.97]	9.78 [4.58–12.30]	0.939 [0.791–0.988]	Excellent
Max coronal plantar angulation (°)	19.83 [10.43–22.44]	16.73 [13.52–24.48]	0.933 [0.707–0.983]	Excellent

ICC, intraclass correlation coefficient; CI, confidence interval; IQR, interquartile range. ^a^ Classification per Koo and Li [22]: ICC > 0.90 = excellent; ICC 0.75–0.90 = good; ICC 0.50–0.75 = moderate; ICC < 0.50 = poor.
